# Tendon elongation in the free tendon is evident in patients with and without persistent muscle weakness following an Achilles tendon rupture

**DOI:** 10.1002/ksa.70445

**Published:** 2026-05-20

**Authors:** Pawel Szaro, Andreas Meunier, Katarzyna Bokwa‐Dabrowska, Katarina Nilsson Helander, Pernilla Eliasson

**Affiliations:** ^1^ Department of Radiology, Institute of Clinical Sciences, Sahlgrenska Academy University of Gothenburg Gothenburg Sweden; ^2^ Department of Radiology Sahlgrenska University Hospital, Region Västra Götaland Gothenburg Sweden; ^3^ Department of Orthopaedics Linköping University Hospital Linköping Sweden; ^4^ Department of Orthopaedics, Institute of Clinical Sciences, Sahlgrenska Academy University of Gothenburg Gothenburg Sweden; ^5^ Department of Orthopaedics Sahlgrenska University Hospital, Region Västra Götaland Gothenburg Sweden; ^6^ Center for Medical Image Science and Visualization (CMIV) Linköping University Linköping Sweden

**Keywords:** Achilles tendon rupture, magnetic resonance imaging, muscle endurance, soleus muscle, tendon elongation

## Abstract

**Purpose:**

Incomplete recovery with persistent muscle weakness is frequently observed following Achilles tendon rupture. The mechanisms for this weakness remain unclear, but tendon elongation has been suggested as a contributing factor. The aim of this study was to compare tendon and muscle morphology in high‐ and low‐functioning patients more than 2 years after non‐surgical treatment of a total Achilles tendon rupture.

**Methods:**

Forty‐six patients underwent screening, including a standardized heel‐rise work test on both legs. Based on this test, a heel‐rise index (HRI) for total muscle work, categorized 29 patients into a low‐ (HRI < 33%) or high‐functioning (HRI > 67%) group. Both groups underwent bilateral magnetic resonance imaging to assess tendon and muscle morphology, and the main variable was tendon elongation.

**Results:**

High‐functioning patients were on average 13 years younger than the low‐functioning patients (*p* < 0.001). Free tendon length was longer on the injured side in both groups, 3.59 and 5.19 cm in the high‐ and low‐functioning group (*p* = 0.053). Tendon cross‐sectional area was significantly larger in the high‐functioning group compared to the low‐functioning group (383% vs. 256% after normalization to the uninjured side, *p* = 0.005). The soleus muscle had notable differences between the groups, as low‐functioning patients had a smaller mediolateral diameter (*p* = 0.002), a more pronounced muscle length difference (*p* = 0.009) and a higher atrophy grading. Additionally, there were significant correlations between age, HRI and tendon size.

**Conclusion:**

Free tendon length after rupture may play a role in muscle weakness. However, tendon elongation does not necessarily lead to low function, as it was also evident in the high‐functioning group. These findings may be important, as they suggest that tendon elongation is not the sole determinant of functional outcome and that other factors may contribute to muscle performance after rupture. Further research is needed on the role of age in muscle function following Achilles tendon ruptures.

**Level of Evidence:**

Level III.

**Abbreviations**
ATRS: Achilles tendon Total Rupture Score
CI: confidence interval
HRI: heel‐rise index
LSI: limb symmetry index
MRI: magnetic resonance imaging

## INTRODUCTION

The Achilles tendon is the largest and strongest tendon in the body, connecting the calf muscle (soleus, gastrocnemius medialis and lateralis muscles) to the calcaneus. It plays a crucial role during locomotion by transmitting forces during walking, running and jumping. Its unique structure, composed of subtendons, allows efficient load transfer, but also results in heterogeneous strain distribution under normal loading conditions [4]. Despite its strength, it is highly prone to injury [29]. Tendon injuries are multifactorial, and total Achilles tendon ruptures most often occur during sporting activities.

A total Achilles tendon rupture is a highly debilitating condition, and the incidence is rising [29], particularly among physically active adults. Despite advances in treatment for Achilles tendon rupture, with comparable outcomes between surgical and non‐surgical treatments [20], many patients fail to achieve full functional recovery [9, 20, 24, 32]. Persistent deficits include reduced restoration of calf muscle strength [23], particularly in plantarflexion and endurance [9]. Tendon elongation has emerged as a key factor influencing recovery after total Achilles tendon rupture [2].

Tendon elongation is defined as an increased distance between tendon ends during healing, and this can continue for up to 6 months post‐rupture, even with surgical treatment [9, 12, 13]. Severe tendon elongation can negatively impact mechanical efficiency, joint mechanics and muscle force generation [30]. This effect is closely linked to muscle adaptations, such as changes in muscle fascicle length, atrophy in the medial gastrocnemius and soleus muscles, accompanied by compensatory hypertrophy of the synergistic muscle, flexor hallucis longus [11]. Prior research has largely focused on muscle weakness and atrophy; the interplay between tendon elongation and functional outcomes remains unclear. Furthermore, it is unknown whether high‐functioning patients also exhibit tendon elongation, which could be compensated for by better muscle adaptation.

This study aimed to compare tendon and muscle morphology in high‐ and low‐functioning patients to determine whether tendon elongation or muscle atrophy differs between these groups following a total Achilles tendon rupture. We hypothesized that tendon elongation would occur after rupture, and that low‐functioning patients would show structural maladaptations, particularly in the soleus muscle.

## MATERIALS AND METHODS

### Study design

**Figure 1:**
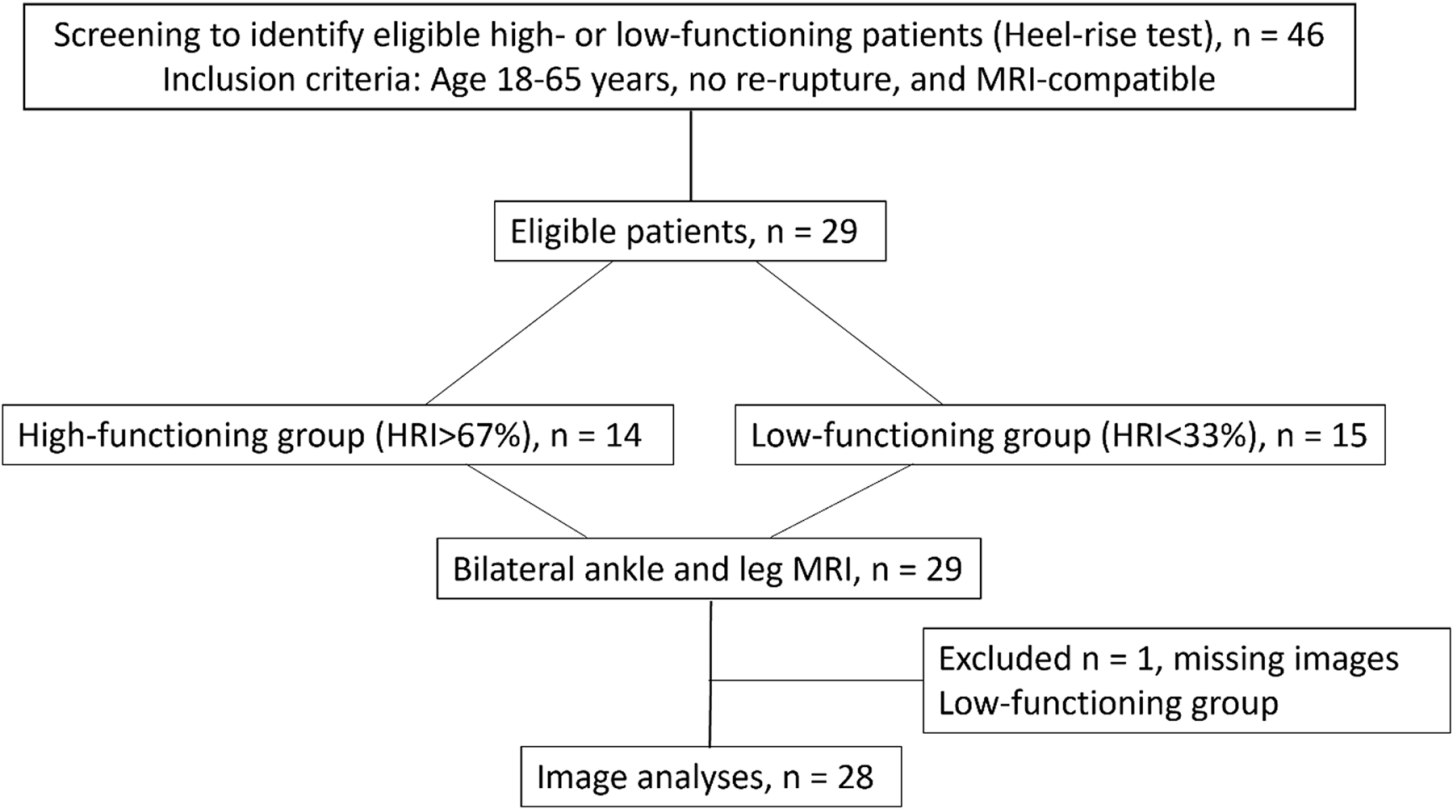
Flowchart illustrating the study setup. A total of 46 patients with a history of non‐surgical treatment for acute total Achilles tendon rupture (≥2 years post‐injury) were screened for eligibility. Inclusion required age 18–65 years, no re‐rupture, and no contraindications for MRI. Based on heel‐rise index (HRI), 29 patients were classified into two groups: low‐functioning (HRI < 33%, *n* = 15) and high‐functioning (HRI > 67%, *n* = 14). MRI, magnetic resonance imaging.

This study was designed as a cross‐sectional follow‐up study with the purpose of comparing high‐functioning patients and low‐functioning patients based on performance in a heel‐rise test. Patients who had undergone non‐surgical treatment for an acute total Achilles tendon rupture at least 2 years prior to the study were invited to participate. They were contacted by phone based on their clinical assessment at 1‐year post‐rupture. All patients were of working age and had received treatment at a single treatment centre, Linköping University Hospital. The non‐surgical treatment was chosen for this study as this is the standard of care at this centre. It consisted of 7 weeks of orthosis use with weight‐bearing on the orthosis as tolerated. All patients were followed for 1‐year post‐injury according to clinical routine by physiotherapists. Ethical approval was obtained from the Swedish Ethical Review Authority (Dnr: 2019‐05547, with an amendment 2024‐04118‐02). The study adhered to the principles of the Declaration of Helsinki, and all participants received oral and written information about the study and provided written informed consent. Inclusion criteria were 18–65 years, no re‐rupture, and no contraindication for magnetic resonance imaging (MRI). A heel‐rise index (HRI) was calculated after an initial screening to quantify the difference between the injured and uninjured sides. Patients with an HRI of <33% or >67% of their uninjured side were included, as our power analysis indicated that we needed approximately 15 individuals per group. To achieve this while maintaining a meaningful distinction between groups, we selected the 33% and 67% cutoffs, which allowed us to form low‐ and high‐functioning groups of sufficient size for statistical power. In total, 29 patients, 2–7 years post‐injury, met these criteria and were allocated to either the low‐functioning group (HRI < 33%, *n* = 15) or high‐functioning (HRI > 67%, *n* = 14). The setup of the study is illustrated in Figure 1.

### Participant screening and assessment

Forty‐six patients participated in a screening with a functional assessment, performed by the principal investigator (P.E.) (Figure 1). This assessment included a muscle endurance test, the heel‐rise work test [26]. The heel‐rise work test was performed bilaterally using a standardized single‐leg standing heel‐rise protocol on a step, as previously described [9]. The test was paced by a metronome, with each concentric and eccentric phase lasting one second. Testing was terminated when the participant discontinued due to fatigue, failed to maintain the pace, or was unable to achieve a heel elevation of at least 5 cm. Data acquisition was performed using MuscleLab® (Ergotest Technology) computer software, and a linear encoder sensor attached to the heel was used for this evaluation. The total number of heel rises, the total distance and maximal heel‐rise height were recorded. For analysis, the total work, calculated as body weight multiplied by total vertical displacement and expressed in joules, was determined. Results were normalized to the contralateral, non‐injured side (total muscle work injured side/total muscle work uninjured side × 100) and expressed as a percentage, a HRI. The assessment also included the Achilles tendon Total Rupture Score, ATRS (for self‐reported perception of outcome), a maximum range of motion test, and a single‐leg vertical jump test. The ATRS ranges from 0 to 100, where 100 is full recovery [22]. The maximum dorsiflexion range of motion was assessed with a weight‐bearing lunge test, so‐called knee‐to‐wall test, measuring the maximum distance between the wall and each foot while the patient could still touch the wall with their knee, without lifting the heel [15]. The patients were also instructed to do a single‐leg vertical jump test [18]. To perform this test, the patient stands on one leg and performs a self‐selected jump as long as possible and then lands on the same leg. They always started with the uninjured side and had a familiarisation attempt on each leg before they performed the jump. A side‐to‐side difference was calculated for all measurements (injured − uninjured side), and a limb‐symmetry index was also calculated (injured/uninjured side × 100).

### MRI protocol

Twenty‐nine patients who were included after the screening were subjected to bilateral MRI to determine structural parameters (length and diameter) in tendon and muscle tissue using a 3 T scanner (Philips, MR systems Ingenia, version 5.9). The scan covered the length of approximately 60 cm, measured from the calcaneal tuberosity upward, and included the middle of the thigh, the entire lower leg and the ankle joint with the calcaneus. Coverage was achieved using contiguous coronal multi‐station acquisitions. Participants were placed in a fixed supine position with limbs close together using rubber bands, and the feet were placed against a plastic foot plate in 90° flexion (anatomical neutral) using a body coil. The radiographer visually confirmed equal contact between the heels and the footplate for both sides. The following MRI protocol was used: A coronal 3D spoiled gradient‐echo acquisition (T1‐type) with water–fat separation was obtained; water‐only images were used for analysis (voxel size 1 × 1 × 3 mm, matrix 512 × 512, TR ~ 4.7 ms, flip angle 10°, slice thickness/spacing 3/3 mm). One patient in the low‐functioning group was later excluded due to unsaved MRI data.

### Raters and image analysis

Two certified musculoskeletal radiologists (P.S. and K.B.‐D.) performed the analysis. Both were blinded to the functional status of the patients. The radiologists independently and in a random order measured the anterior–posterior and mediolateral diameters of the Achilles tendon, as well as the soleus and the medial and lateral heads of the gastrocnemius on both the injured and uninjured sides. Raters used PostDicom© on a dedicated radiological workstation equipped with diagnostic‐grade monitors. The radiologists independently selected the cross‐sectional images for measurements.

**Figure 2:**
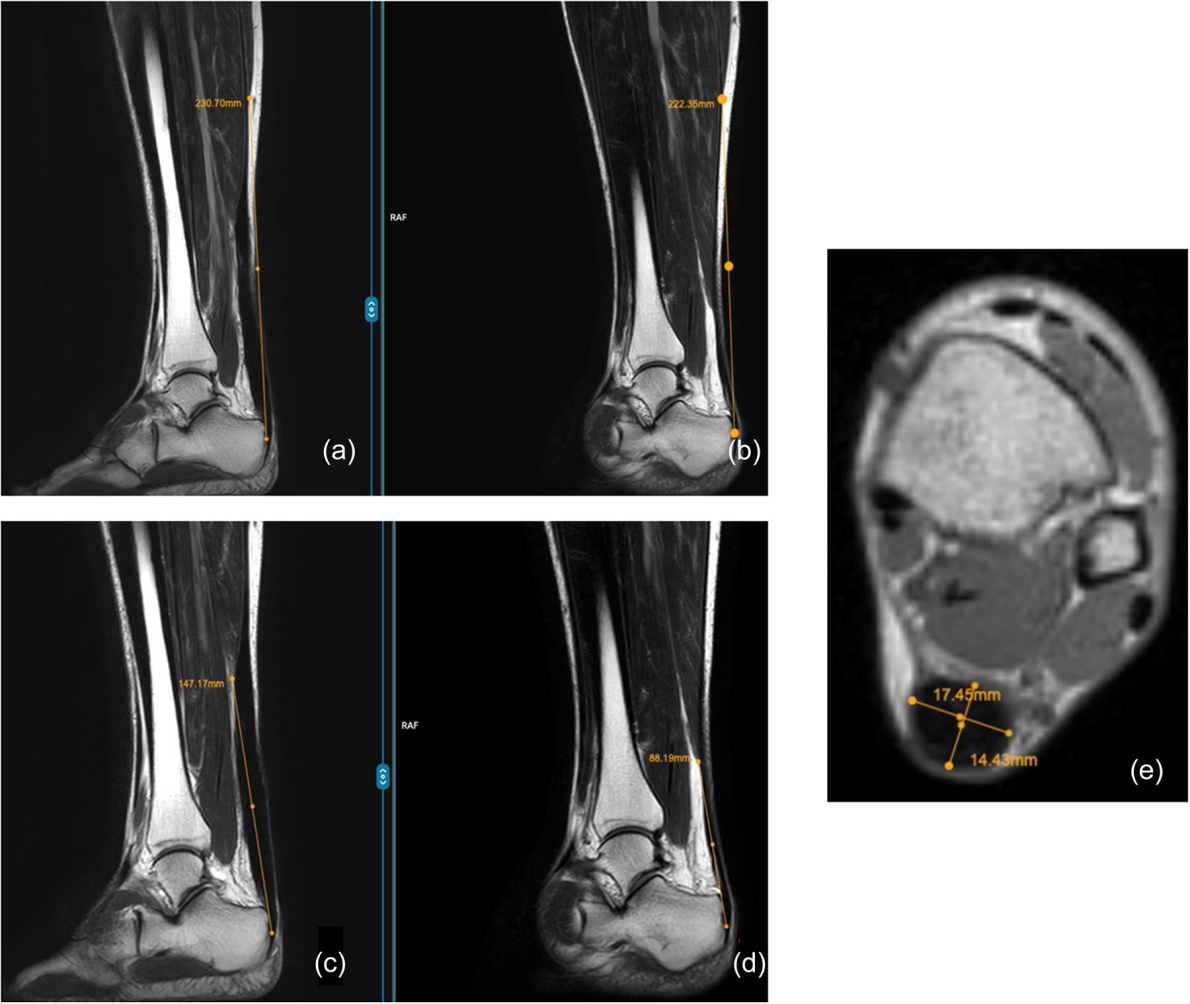
Sagittal and transverse sections of the Achilles tendon with measurements (T1‐weighted images). (a and b) Sagittal sections showing measurements of total tendon length: (a) injured side and (b) non‐injured side. (c and d) Sagittal sections showing measurements of free tendon length: (c) injured side and (d) non‐injured side. (e) Transverse section of the injured leg with measurements of mediolateral and anterior–posterior diameters.

Radiologists measured the length of the total tendon from the calcaneal tuberosity to the medial head of the gastrocnemius muscle [3] and the free tendon defined as the distance from its attachment at the calcaneal tuberosity to the inferior margin of the soleus muscle [8, 31] (Figure 2). Tendon elongation was defined as the difference in tendon length between the injured and uninjured sides [27]. The anterior–posterior and mediolateral diameters of the Achilles tendon were measured on the transverse cross‐section at the point of maximum thickness (Figure 2). These measurements were used to calculate an estimated cross‐sectional area (π × anterior–posterior × mediolateral/4).

**Figure 3:**
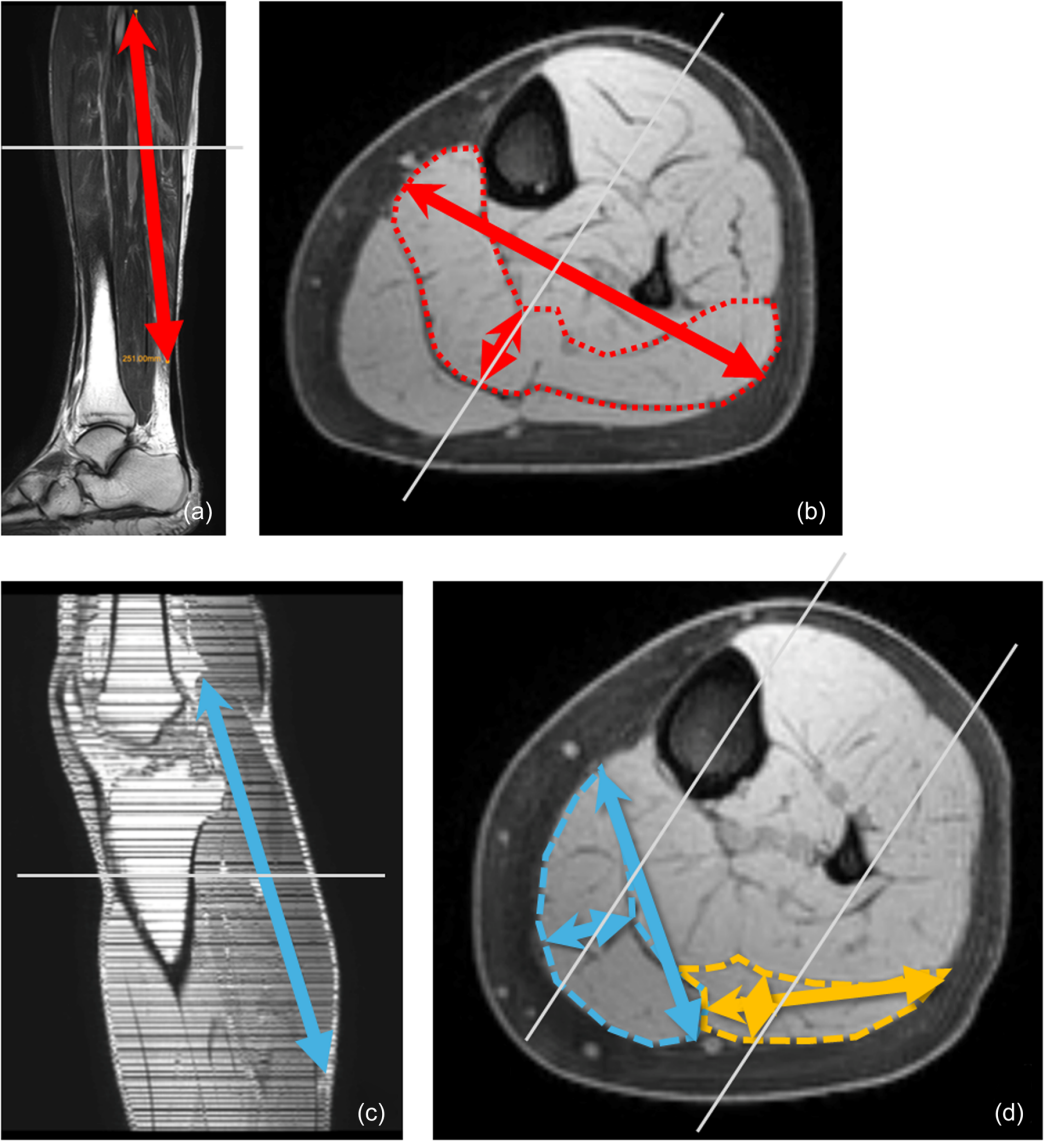
Sagittal and transverse sections of the soleus and gastrocnemius muscles. (a) Sagittal section showing the soleus muscle, with its length indicated by a red arrow. (b) Transverse section with the soleus outlined by a dashed red line; arrows indicate measurements of mediolateral (ML) and anterior–posterior (AP) diameters. (c) Sagittal section showing the medial gastrocnemius muscle, with its length indicated by a blue arrow. (d) Transverse section with the gastrocnemius heads outlined: medial head (blue dashed line) and lateral head (yellow dashed line); arrows indicate measurements of ML and AP diameters. White lines denote the levels of the corresponding slices.

Soleus length was defined as the maximum distance between the most superior and inferior points of the muscle belly in the sagittal section (Figure 3) [7]. Soleus thickness was measured as the greatest anterior–posterior diameter in the transverse section, and soleus breadth was defined as the maximum mediolateral diameter in the transverse section. The medial head of the gastrocnemius was defined as follows: length was the maximum distance between the most superior and inferior points of the muscle belly in the sagittal plane (Figure 3). The thickness was measured as the greatest anterior–posterior diameter in the transverse section, and breadth was defined as the maximum mediolateral diameter in the transverse section. These measurements were used to calculate an estimated maximal cross‐sectional area (π × anterior–posterior × mediolateral/4) and muscle volume (cross‐sectional area × length). The size of the lateral head was only evaluated for the anterior–posterior and mediolateral diameter, as the length measurement was unfortunately not possible due to the imaging acquisition.

Raters also evaluated the grade (0–4) of soleus and gastrocnemius atrophy on T1‐weighted images, assessing the presence of intramuscular fat and comparing the muscles to the anterior muscle group. Atrophy was assessed using the muscle‐wasting criteria described by Goutallier for rotator cuff pathology [28]. The Goutallier classification quantifies the degree of fatty atrophy after a tendon tear and is based on the percentage of fatty degeneration in the affected muscle, graded from 0 to 4, with higher grades indicating worse functional outcomes. The grades are defined as follows: 0—Normal muscle, 1—Some fatty streaks, 2—Less than 50% fatty muscle atrophy, 3—50% fatty muscle atrophy and 4—Greater than 50% fatty muscle atrophy. This score has been strongly correlated with quantitative fat‐fraction maps in muscle compartments of the lower leg [1].

### Statistical analysis

The primary outcome variable was tendon elongation, and based on this, power calculations estimated that a sample of *N* = 12 per group would be required to detect a 4 mm difference with 80% power (SD = 3 mm, 2‐tailed *α* = 0.05). This corresponds to an effect size (Cohen's *d*) of approximately 1.33, indicating a large effect size. Statistical analyses were performed using SPSS, and graphs were created using Graphpad Prism. Student's *t* test was used for comparisons between groups and paired *t* test for comparisons between the injured and uninjured side, provided that the assumption of normality (Shapiro–Wilk test) and homogeneity of variance (Levene's test) were met. Variables that did not meet the normality assumption were analyzed using the Mann–Whitney *U* test. As the groups differed significantly in age, additional age‐adjusted analyses were conducted. We performed an ANCOVA with age included as a covariate to evaluate between‐group differences in the primary outcome, tendon elongation, as well as secondary outcomes that demonstrated significant group differences in the initial comparisons (CSA LSI and soleus length LSI). Between‐group differences in categorical variables were analyzed using the Pearson chi‐square test. Correlation analyses were also performed between HRI, age and structural properties (e.g., tendon diameter and muscle length) using Pearson correlations. The correlations between HRI and structural properties were pre‐specified, while the correlations with age were exploratory. Inter‐ and intra‐rater reliability was assessed for MRI measurements, and an intraclass correlation coefficient was calculated. Intra‐rater reliability was assessed in a subset of six randomly chosen patients using repeated MRI measurements performed by the same observer. The measurements on the MRI for Achilles tendon diameters and soleus morphology showed excellent reproducibility, according to Koo and Li [16]. The final results from all MRI analyses are presented as mean values between raters.

## RESULTS

The groups were similar in height, weight and body mass index (Table 1). There was a clear age difference between the two groups, with the high‐functioning group being significantly younger (42.6 vs. 56.0 years, *p* < 0.001). ATRS was better in the high‐functioning group by 15 points (90.6 vs. 75.8, *p* = 0.042). LSI for the jump test was 99% in the high‐functioning group versus 93% in the low‐functioning group (*p* = 0.058), while ankle range of motion was similar between the groups (Table S1).

**Table 1:** Descriptive data (mean ± SD) for the high‐ and low‐functioning group.

	High‐functioning group	Low‐functioning group	*p*
Age, year	42.6 ± 6.70	56.0 ± 6.43	<0.001
Height, cm	177 ± 6.50	178 ± 5.82	0.785
Weight, kg	84.7 ± 10.0	85.0 ± 14.4	0.952
BMI	27.1 ± 3.80	26.8 ± 4.26	0.880
HRI, %	84.6 ± 13.8	14.9 ± 11.4	<0.001
ATRS	90.6 ± 16.2	75.8 ± 20.3	0.042

*Note*: *p* values are derived from Student's *t* test between the groups.

Abbreviations: ATRS, Achilles tendon Total Rupture Score; BMI, body mass index; HRI, heel‐rise index.

### MRI findings

MRI revealed no tendon defects in any of the patients. Signal alterations in the Achilles tendon (higher signal than normal) were observed in three patients (two high‐functioning and one low‐functioning), and Kager's fat pad changes were present in 12 patients (six in each group). Plantar fascia and calcaneal bursa were normal in all patients.

### Achilles tendon structure

**Figure 4:**
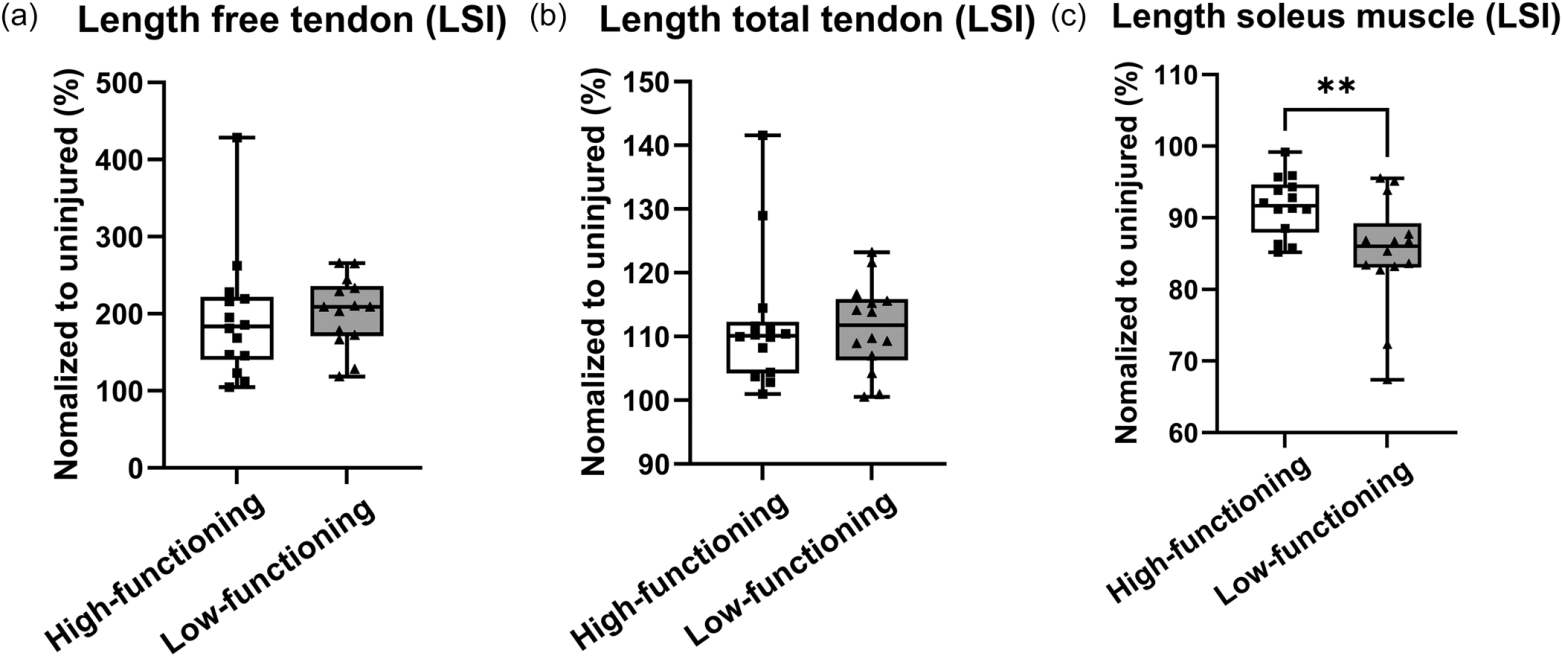
Box plots of limb symmetry index (LSI) for structural properties of the Achilles tendon and soleus muscle in high‐ and low‐functioning patients. (a) Free tendon elongation, (b) total tendon elongation and (c) soleus muscle length in high‐functioning (white) and low‐functioning (grey) patients. Only the soleus muscle length LSI showed a significant difference between the two groups. Each data point represents one individual.

**Figure 5:**
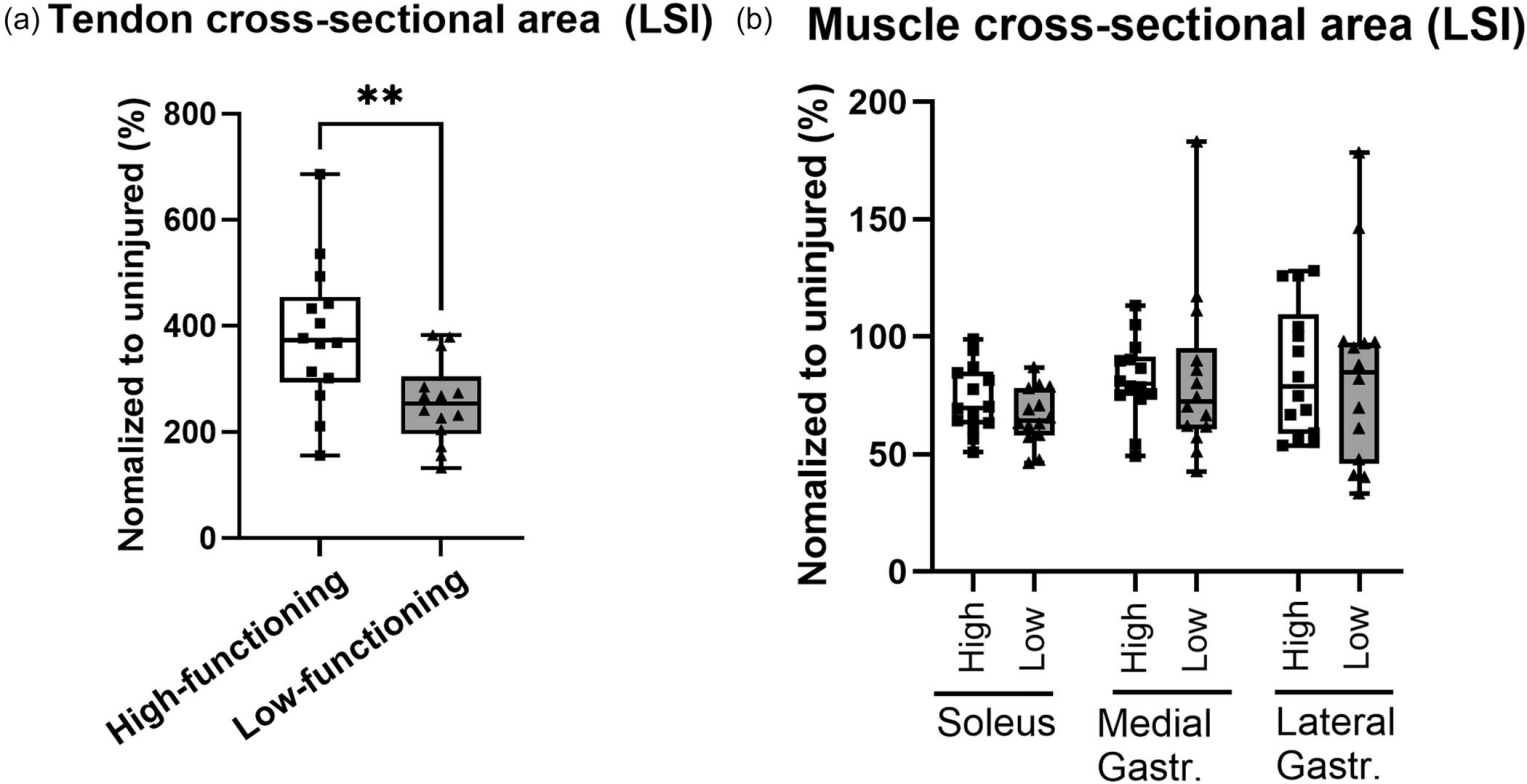
Box plots for structural properties for the tendon and muscles in high‐ and low‐functioning patients. (a) Limb symmetry index (LSI) for tendon cross‐sectional area (CSA) and (b) muscle CSA for the individual calf muscles, soleus, medial gastrocnemius (GC med) and lateral gastrocnemius (GC lat) in high‐ (white) and low‐functioning (grey) patients. Each data point represents one individual. Tendon CSA presented significant differences between the two groups, but not the muscle properties.

The free tendon length was longer on the injured side compared to the uninjured side in both groups (Table 2), with 5.2 cm elongation in the low‐functioning group versus 3.6 cm in the high‐functioning group (*p* = 0.053, Figure 4), though LSI was similar (*p* = 0.323, Table 2). Total tendon elongation was smaller (~2.5 cm) and did not differ between the two groups (*p* = 0.779). Tendon diameters and cross‐sectional area were larger on the injured side compared to the uninjured side in both groups (Table 2). The side‐to‐side difference for tendon diameter was 0.7 cm in the high‐functioning group and 0.5 cm in the low‐functioning group. The side‐to‐side difference in cross‐sectional area was significantly greater in the high‐functioning group (383% vs. 256%, *p* = 0.005, Figure 5). Adjusting for age, using an ANCOVA, did not change the outcomes, and the results remained consistent with the original analyses. The result for the primary variable tendon elongation was *p* = 0.148 with the ANCOVA, and for the LSI *p* = 0.894. The corrected results for the side‐to‐side difference in cross‐sectional area were *p* = 0.003.

**Table 2:** Structural data from MRI on the uninjured and injured side, and the difference between the sides, are expressed in absolute values and LSI.

		High‐functioning group				Low‐functioning group				
		Uninjured	Injured	Difference	LSI %	Uninjured	Injured	Difference	LSI %	LSI: *p*
Achilles tendon	AP diameter, cm	0.57 ± 0.12	1.32 ± 0.16	0.74 ± 0.20	240 ± 56.0	0.69 ± 0.14	1.22 ± 0.18	**0.54** ± **0.21**	184 ± 40.1	0.006
	ML diameter, cm	1.18 ± 0.15	1.81 ± 0.18	0.63 ± 0.24	156 ± 24.4	1.29 ± 0.14	1.75 ± 0.16	**0.46** ± **0.17**	137 ± 14.8	0.018
	Est. CSA, cm^2^	0.54 ± 0.18	1.90 ± 0.37	1.35 ± 0.39	383 ± 136	0.70 ± 0.20*	1.68 ± 0.30	**0.98** ± **0.36**	256 ± 78.6	0.005
	Free length, cm	4.29 ± 1.55	7.88 ± 3.10	3.59 ± 2.29	194 ± 81.9	5.78 ± 2.14*	11.0 ± 2.17*	5.19 ± 1.87	202 ± 45.4	*0.323*
	Total length, cm	21.0 ± 2.80	23.3 ± 2.38	2.35 ± 1.78	112 ± 10.8	22.6 ± 2.16	25.1 ± 2.78	2.59 ± 1.54	111 ± 6.96	*0.779*
Soleus	AP diameter, cm	2.43 ± 0.30	1.87 ± 0.31	−0.57 ± 0.33	77.2 ± 12.6	2.14 ± 0.19*	1.63 ± 0.29	−0.51 ± 0.31	76.7 ± 13.5	0.917
	ML diameter, cm	9.24 ± 0.63	8.73 ± 0.69	−0.51 ± 0.52	94.7 ± 5.64	8.78 ± 0.69	7.61 ± 0.88*	**−1.18** ± **0.58**	86.6 ± 6.92	0.002
	Est. CSA, cm^2^	17.7 ± 2.86	12.8 ± 2.34	−4.90 ± 2.80	73.3 ± 14.3	14.8 ± 2.03*	9.73 ± 2.00*	−5.07 ± 2.08	66.1 ± 11.9	0.160
	Est. Volume, cm^3^	604 ± 106	399 ± 74.0	−205 ± 90.7	67.0 ± 11.9	481 ± 83.4*	269 ± 69.1*	−212 ± 78.3	56.4 ± 12.3	0.029
	Length, cm	34.1 ± 1.93	31.4 ± 2.69	−2.80 ± 1.33	91.7 ± 4.11	32.4 ± 2.69	27.5 ± 3.56*	**−4.85** ± **2.60**	85.0 ± 7.80	0.009
Medial gastroc.	AP diameter, cm	2.43 ± 0.31	2.18 ± 0.42	−0.25 ± 0.56	91.2 ± 20.9	2.28 ± 0.43	1.89 ± 0.45	−0.39 ± 0.61	85.6 ± 26.5	0.541
	ML diameter, cm	7.12 ± 0.60	6.46 ± 0.82	−6.64 ± 5.45	90.6 ± 7.72	7.14 ± 0.95	6.63 ± 0.71	−5.07 ± 7.62	93.7 ± 10.7	0.381
	Est. CSA, cm^2^	13.6 ± 2.30	11.0 ± 2.02	−2.65 ± 2.74	81.9 ± 17.2	12.9 ± 3.68	9.86 ± 2.69	−3.05 ± 4.42	82.2 ± 35.9	0.969
	Est. Volume, cm^3^	330 ± 56.3	270 ± 55.6	−60.2 ± 53.3	82.5 ± 17.0	284 ± 95.9	233 ± 75.1	−51.3 ± 111	88.6 ± 41.7	0.659
	Length, cm	24.6 ± 2.33	23.8 ± 2.10	−0.81 ± 0.98	96.8 ± 3.87	23.8 ± 2.11	22.93 ± 1.82	−0.88 ± 1.69	96.5 ± 6.68	0.903
Lateral gastroc.	AP diameter, cm	1.69 ± 0.45	1.45 ± 0.44	−0.24 ± 0.38	88.0 ± 20.8	1.74 ± 0.35	1.60 ± 0.44	−0.15 ± 0.52	94.8 ± 34.2	0.532
	ML diameter, cm	5.59 ± 0.69	5.33 ± 0.76	−0.26 ± 0.62	95.7 ± 11.2	5.58 ± 0.64	4.74 ± 0.90	−0.84 ± 1.04	86.0 ± 19.7	0.119
	Est. CSA, cm^2^	7.50 ± 2.45	6.23 ± 2.56	−1.27 ± 1.98	85.4 ± 27.6	7.72 ± 2.08	6.08 ± 2.36	−1.64 ± 2.74	84.0 ± 41.0	0.911

*Note*: Mean ± SD bold indicates a significant difference between the groups in absolute values. *p* values derive from Student's *t* test between the groups, except for the *p* values in italics, which derive from a Mann–Whitney *U* test.

Abbreviations: AP, anterior‐posterior; CSA, cross‐sectional area; LSI, limb symmetry index (injured side/uninjured side × 100, expressed as a percentage); ML, mediolateral; MRI, magnetic resonance imaging.

^a^ Indicates a significant difference between the high‐ and low‐functioning groups on the equivalent side.

### Muscle properties

The soleus muscle had fatty degeneration in all but one patient in the low‐functioning group, while four patients in the high‐functioning group demonstrated no fatty degeneration (Table 3, *p* = 0.054). Soleus muscle volume LSI was smaller in the low‐functioning group (57% versus 67%, *p* = 0.029, Table 2), and muscle length was significantly shortened (4.9 cm vs. 2.8 cm, *p* = 0.014, Figure 4). The LSI for muscle length was also smaller in the low‐functioning group (85% vs. 92%, *p* = 0.009). Soleus muscle diameter was smaller compared to the uninjured leg. Only mediolateral diameter was significantly smaller in the low‐functioning group (87% vs. 95%, *p* = 0.002). Soleus muscle cross‐sectional area showed no difference between the two groups (Figure 5).

**Table 3:** Atrophy grading of muscles.

	Atrophy grading	High‐functioning	Low‐functioning	*p* *
Soleus	0	4	1	0.054
	1	10	9	
	2	0	4	
	3	0	0	
Medial gastroc.	0	14	11	0.067
	1	0	3	
	2	0	0	
	3	0	0	
Lateral gastroc.	0	14	11	0.067
	1	0	3	
	2	0	0	
	3	0	0	

* *p* values derive from the Pearson chi‐square test.

The gastrocnemius muscles showed minimal differences between groups, with three patients exhibiting some atrophy in the medial gastrocnemius and three in the lateral gastrocnemius muscle (all in the low‐functioning group, *p* = 0.067, Table 3). Medial gastrocnemius volume LSI was also similar in the two groups (89% vs. 83%, *p* = 0.659, Table 2). Volume estimation for the lateral gastrocnemius muscle was not calculated, as length measurements were not possible to perform. Lateral gastrocnemius cross‐sectional area LSI showed no difference between the two groups (84‐86% in both groups, *p* = 0.911).

### Correlations between HRI or age structural properties

**Figure 6:**
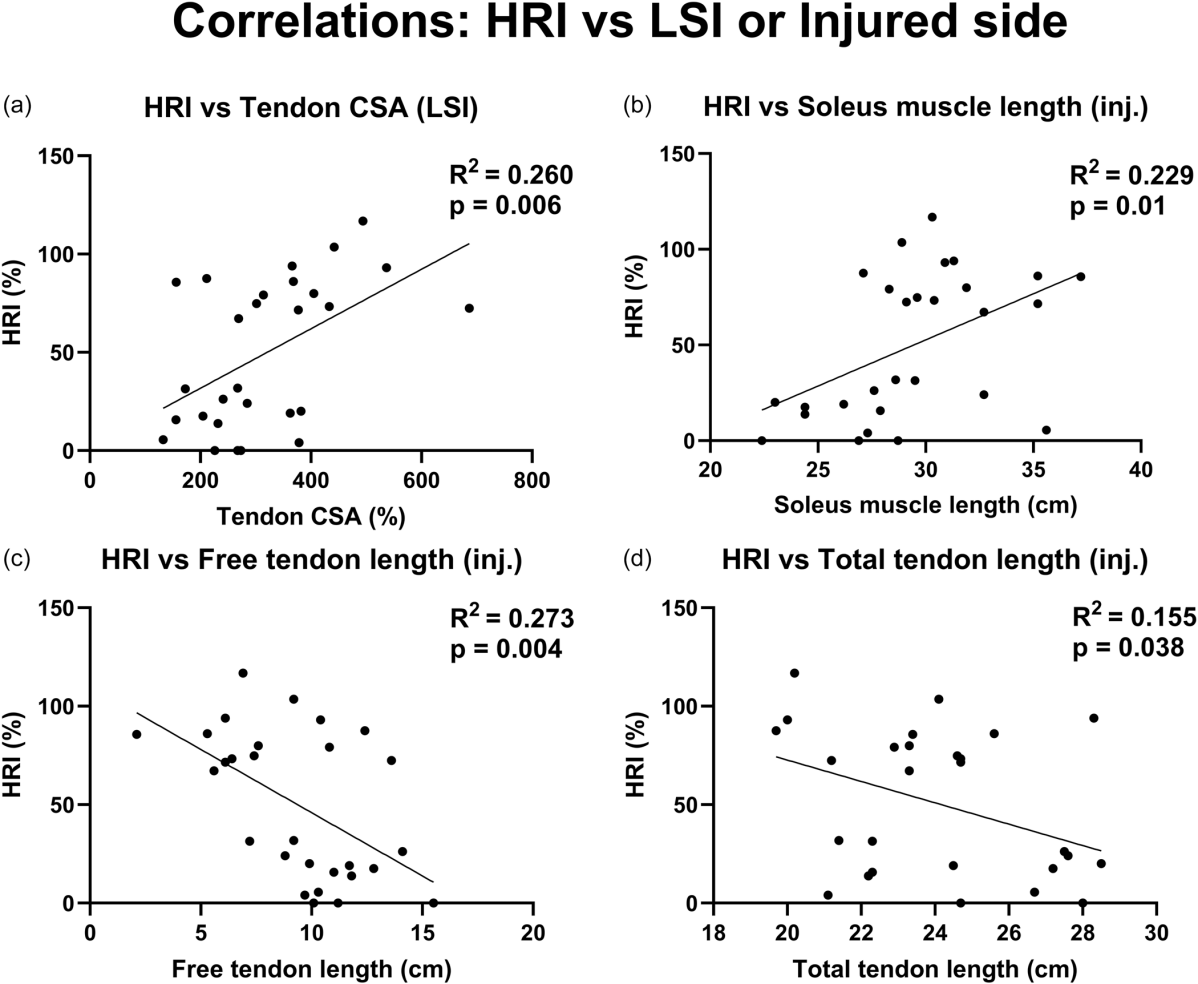
Correlation analyses between heel‐rise index (HRI) and structural properties for the tendon and muscle. (a) HRI versus limb symmetry index (LSI) for tendon cross‐sectional area (CSA), (b) soleus muscle length on the injured side, (c) free tendon length on the injured side and (d) total tendon length on the injured side. All significantly correlated (using Pearson's correlation analysis) with HRI, with a positive correlation for tendon CSA and soleus length, and a negative correlation for tendon length (free and total).

**Figure 7:**
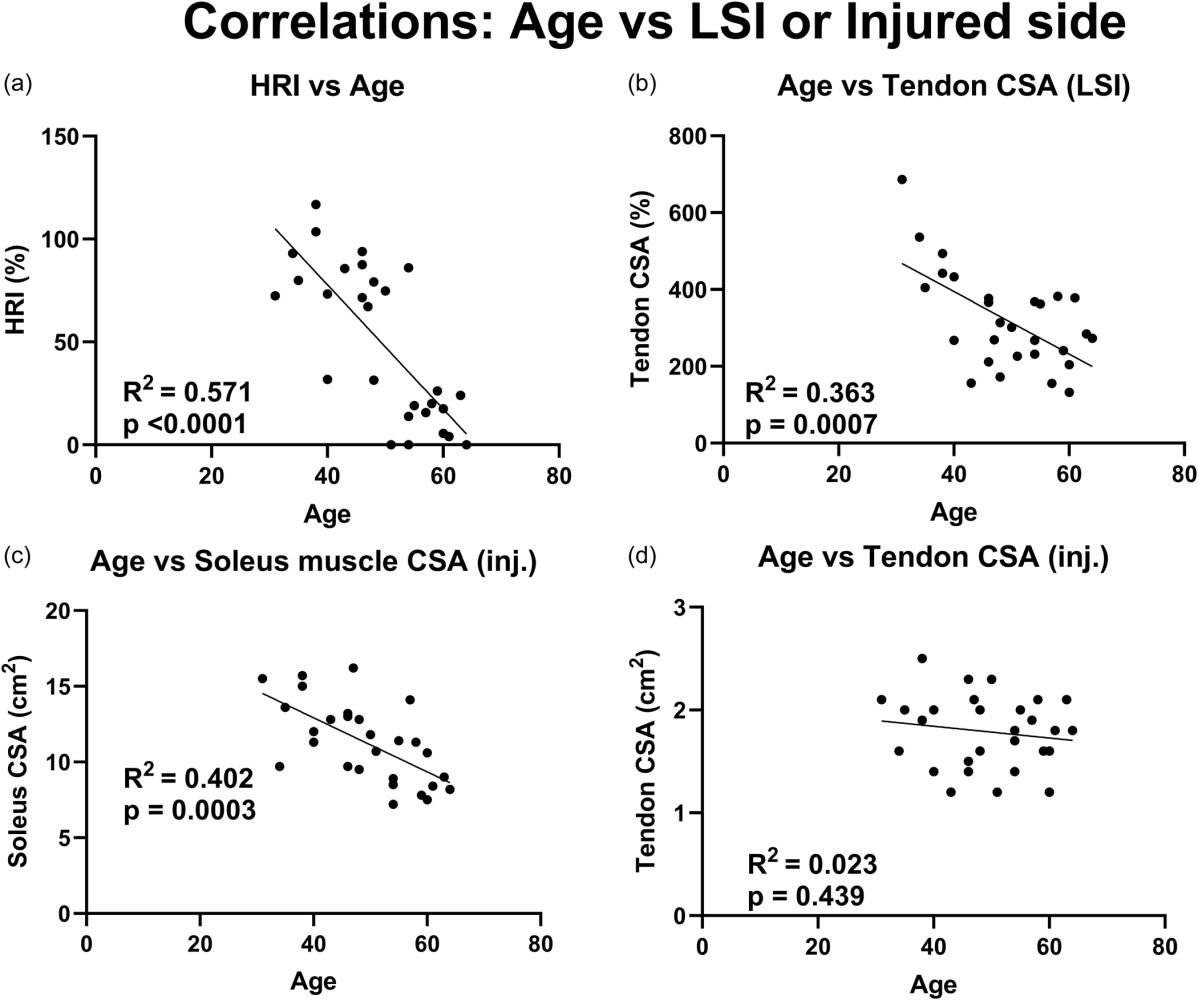
Correlation analyses between heel‐rise index (HRI), age and structural properties in the tendon and muscle. (a) HRI versus age, (b) age and limb symmetry index (LSI) for tendon cross‐sectional area (CSA), (c) soleus muscle CSA on the injured side and (d) tendon CSA on the injured side. All correlations except for tendon CSA on the injured side were significant (using Pearson's correlation analysis).

**Figure 8:**
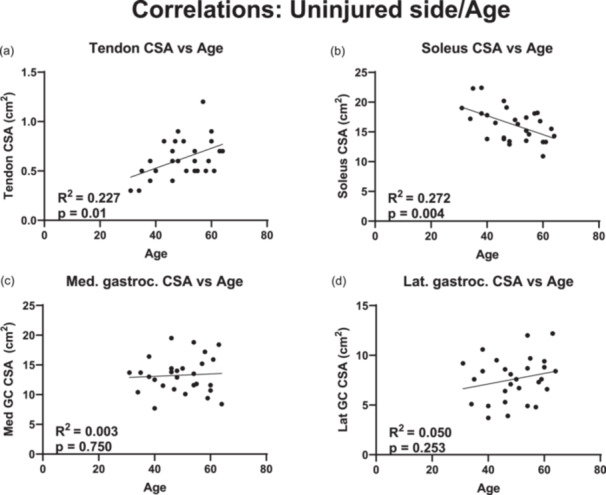
Correlation analyses between age and structural properties in the tendon and muscle. (a) Age versus tendon cross‐sectional area (CSA) on the uninjured side, (b) age versus soleus muscle CSA on the uninjured side, (c) age versus medial gastrocnemius muscle CSA on the uninjured side and (d) age versus lateral gastrocnemius muscle CSA on the uninjured side. Tendon and soleus CSA showed significant correlations with age, but there was no correlation found for the gastrocnemius muscles (using Pearson's correlation analysis).

Lower HRI (worse function) correlated with smaller Achilles tendon cross‐sectional area (LSI) (*p* = 0.006, Figure 6). There was also a correlation between lower HRI and longer free tendon length on the injured side (*p* = 0.004), and total tendon length (*p* = 0.038). Higher HRI (better function) correlated with longer soleus length on the injured side (*p* = 0.01) as well as higher soleus length LSI and volume (*R* ^2^ = 0.213, *p* = 0.013 and *R* ^2^ = 0.157, *p* = 0.037, respectively). No correlation was found between HRI and tendon length LSI (free or total). Higher age correlated with worse HRI (*p* < 0.0001), smaller Achilles tendon cross‐sectional area LSI (*p* = 0.0007), and smaller soleus cross‐sectional area on the injured side (*p* = 0.0003, Figure 7). No correlation was found for Achilles tendon cross‐sectional area on the injured side (*p* = 0.439). Correlations were also found between age and tendon and muscle properties on the uninjured side. Higher age correlated to larger Achilles tendon cross‐sectional area (*p* = 0.01), and smaller soleus muscle cross‐sectional area (*p* = 0.004), but not to the cross‐sectional area of the gastrocnemius muscles (Figure 8).

### Reliability in MRI evaluation

Intra‐rater reliability for MRI measurements demonstrated high repeatability, with intraclass correlation coefficients indicating excellent agreement for Achilles tendon diameter, free tendon length, soleus muscle length, and gastrocnemius muscle length (intraclass correlation coefficient values = 0.917, CI: 0.976–0.998, 0.978, CI: 0.919–0.998, 0.994, CI: 0.955–0.999, and 0.949 CI: 0.921–0.998). Anterior–posterior diameter of the soleus in the midsagittal plane showed good agreement (intraclass correlation coefficients = 0.781, CI: 0.133–0.970). Interrater reliability was good to excellent for all variables, with intraclass correlation coefficient values for Achilles tendon between 0.864 and 0.918 and for soleus muscle 0.875–0.895 (Table S2).

## DISCUSSION

This study aimed to compare morphological differences in the tendon and muscle in high‐ and low‐functioning patients to investigate whether tendon elongation or muscle atrophy differed following a total Achilles tendon rupture. Our findings reveal several key differences between the two groups, which may provide insights into the factors influencing recovery and rehabilitation. (1) tendon elongation is evident in both high‐ and low‐functioning patients with most pronounced effect in the free tendon length, (2) diminished muscle function is associated with smaller tendon cross‐sectional area and shorter soleus muscle, and (3) age correlates with both muscle deterioration and lower functional recovery.

### Tendon elongation alone does not account for functional differences; soleus muscle morphology is a stronger determinant of long‐term performance

Clinical understanding of Achilles tendon rupture recovery suggests that tendon morphology and muscle adaptations are key determinants of long‐term function. Tendon elongation has previously been discussed in conjunction with poor functional outcomes, albeit with mixed outcomes [2, 9, 12, 25, 27]. Longer free tendon length and reduced soleus muscle capacity have been linked to persistent weakness [12, 30], while a larger tendon cross‑sectional area has been associated with more favourable outcomes [24, 34]. Consistent with this, we observed that longer free tendon length was correlated with poorer functional performance. However, we also observed that tendon elongation was pronounced in the high‐functioning group, with no significant difference between the two groups. The discrepancy between tendon length, tendon elongation and functional performance could imply that patients with initially longer free tendons are more susceptible to poor functional recovery after a total Achilles tendon rupture, or that there is a physiological limit to free tendon length. More longitudinal studies may establish a causal association, but this suggest that elongation alone may not fully explain persistent deficits. Other differences in tendon structure were, on the other hand, seen between groups. The high‐functioning group had a larger Achilles cross‐sectional area, which aligns with previous reports suggesting tendon cross‐sectional area, early after rupture, as a predictor of outcome [24, 34]. Clinically, this may reflect improved load‐bearing capacity of the tendon, better enabling patients to regain function. Yet, to our knowledge, there is currently no published minimal clinically important difference for Achilles tendon cross‐sectional area after tendon rupture.

Muscle adaptations followed somewhat expected patterns as well. The soleus muscle in the low‐functioning group had a more pronounced reduction in muscle length, while gastrocnemius heads showed minimal between‐group differences. This is consistent with the suggested vulnerability of the soleus due to its unipennate architecture and its primary insertion into the free tendon [12]. Because of this anatomical relationship, changes in tendon length may disproportionately impair soleus function, which is critical for push‐off strength and endurance. For clinicians, this reinforces the importance of evaluating not only tendon structure but also muscle morphology when assessing recovery potential and rehabilitation, as soleus muscle morphology may be directly related to push‐off capacity.

### Age‐related effects on muscle function, tendon structure, and recovery

Age is known to influence intact tendon structure and muscle adaptation, and has previously been linked to poorer functional outcomes after Achilles tendon rupture [6]. So perhaps not so surprisingly, our results align with this. Our results show that increasing age was associated with reduced muscle function (specifically, lower total muscle work and smaller soleus muscle size), but we found no correlation between age and tendon elongation. On the uninjured side, older age tended to associate with larger Achilles tendon cross‐sectional area but smaller soleus cross‐sectional area, whereas gastrocnemius size showed no age association. These characteristics may reflect tissue degeneration, but they could also reflect natural age‐related tissue changes.

Together, these findings imply that older patients may have a diminished capacity for muscle hypertrophy or exhibit physiological constraints affecting muscle remodelling after rupture. Consequently, rehabilitation strategies might need adjustment, potentially emphasizing slower progression, targeted soleus strengthening, or surgical approaches to mitigate further compromise of an already atrophic muscle. Prospective studies are needed to clarify whether age‐related muscle limitations or tendon changes primarily drive the reduced function. Nevertheless, our data indicate that interventions explicitly targeting soleus recovery may be particularly beneficial in older patients.

### Interindividual anatomical variability, particularly in free tendon length, may influence pathology and outcomes

Structural differences were also noted between the two groups in the uninjured tendon. The low‐functioning group displayed a larger Achilles tendon cross‐sectional area and longer free tendon. The variability in free tendon length may be an important anatomical factor influencing both injury risk and post‐injury outcome, as longer free tendon length has been associated with tendinosis [8]. Therefore, some between‐group differences may stem from inherent anatomical variation or age‐related remodelling rather than injury‐induced changes alone. Therefore, contralateral comparisons should be interpreted with caution, and anatomical variability should be considered when guiding clinical decisions.

### Free tendon elongation is mechanically more consequential than total tendon elongation and correlates more strongly with functional deficits

To better understand the relationship between tendon remodelling and function, we examined elongation in both free and total tendon segments. Our results revealed that free tendon elongation was substantially greater than total tendon elongation, approximately a doubling in length compared with a 12% increase in total tendon length. Moreover, free tendon elongation demonstrated stronger associations with functional deficits, including reduced heel‐rise height and lower limb symmetry index, whereas correlations with total tendon elongation were weaker. These findings align with previous research [2, 12, 14, 25]. Previous research on surgically treated rupture has shown that the free tendon elongation is evident as early as 1 week post‐rupture (~1.7/1.3 cm), with no further elongation over time, in contrast to the total tendon length, which was unchanged at 1 week but elongated over time [12]. Rosso et al. showed approximately 2 cm length difference in total tendon length, which aligns with our results [25]. Along with our results, previous studies have shown associations between free tendon length, HRI and deficits in heel‐rise height [5, 27]. Other studies have found associations with total tendon length, which we did not [30]. Although methodological differences across studies complicate comparisons, our results reinforce the importance of distinguishing tendon segments when assessing elongation. Given its stronger relationship with function, free tendon length should be prioritized in research protocols aiming to quantify tendon healing and mechanical consequences. This may help identify patients at higher risk of long‐term deficits.

### Soleus muscle shortening and atrophy are central features of long‐term deficit and are more pronounced in low‐functioning patients

Fewer studies have investigated muscle shortening. We demonstrated that both groups had patients with soleus atrophy and fatty infiltration, with more severe changes in the low‐functioning group. Svensson et al. demonstrated that surgically treated low‐functioning patients had more pronounced soleus muscle shortening compared to the gastrocnemius muscle, along with shorter muscle fibres [30], aligning with our findings in the low‐functioning group. That study, however, only included patients with low function. Our results also showed reduced muscle length and sometimes atrophy among high‐functioning patients. This highlights that even high‐functioning individuals may experience structural muscle changes. Heikkinen et al. have previously shown that soleus atrophy is common after non‐surgical treatment [10], and even surgically treated patients can have a reduction of 11%–15% in muscle cross‐sectional area after an Achilles tendon rupture [11, 19]. In our cohort, the reduction in soleus cross‐sectional area was more substantial, which may reflect the chronic stage of evaluation or differences in healing mechanisms with the non‐surgical treatment. The reduction in the gastrocnemius medialis and lateralis is more in line with the results in a recent randomised controlled trial [12]. This study also showed that the free fat fraction was higher on the injured side in all muscles, even after 1 week, with no apparent sign of improvement over time. The reduction in muscle dimensions likely reflects a combination of muscle fibre loss and fatty infiltration, with an increase in the fat component within the muscle in the initial phase, followed by a decrease in dimensions and volume. This suggests that rehabilitation should possibly prioritize restoring soleus muscle architecture and strength.

### Low‐functioning patients exhibit clinically meaningful differences in patient‐reported outcomes compared to high‐functioning patients

The ATRS in our study was on average 15 points lower in the low‐functioning group, a difference considered clinically significant and falls within the range described in previous studies [9, 20, 21, 24]. Half of the patients in the low‐functioning group scored below the patient acceptable symptom score of 75 points [17], whereas only one high‐functioning patient fell below this threshold (this patient also experienced pain occasionally in the tendon).

Nearly half of the patients demonstrated abnormalities in Kager's fat pad, an observation that was not anticipated in the study design. The thickness of Kager's fat pad has previously been shown to be larger on the injured side compared to the uninjured side in a retrospective study, but with no correlations on ATRS results [33]. Hence, the functional impact of these changes remains unclear and warrants further investigation. ATRS differences confirm the validity of functional grouping and highlight the need to monitor patient‐reported outcomes alongside structural measures.

Several limitations of this study should be acknowledged. Due to its cross‐sectional design, the findings in this study reflect associations rather than causal relationships. As causality cannot be inferred, future longitudinal studies would be needed to clarify the temporal sequence of changes in tendon structure and function. Moreover, muscle function was assessed solely with the heel‐rise test; no measurements of maximal plantar flexion strength were included. Because the heel‐rise test is effort‐dependent, factors such as patient motivation may have influenced performance/variability. Additionally, no biomechanical gait analysis was performed. The relatively small sample size may affect the generalizability of our findings, and secondary variables could be underpowered. Our cross‐sectional study design did not include an intermediate group, which could have offered another perspective, and we were unable to control for the rehabilitation process after injury. The rehabilitation is likely to vary between patients, and this may have influenced variability in recovery. Patients in the low‐functioning group may also have relied more on their uninjured limb, potentially exaggerating side‐to‐side differences. All patients received non‐surgical treatment, and therefore, the findings may not be generalized to surgically treated populations, in whom tendon healing patterns and scarring differ. Although the latest randomized controlled trial comparing surgical and non‐surgical treatment did not report differences in ATRS or functional test [20]. However, our results still provide important insight into the natural course and compensation mechanisms after non‐surgical treatment.

Regarding morphological measurements, tendon diameters and the estimated cross‐sectional area were assessed, but the actual cross‐sectional area was not directly measured. This limitation may have reduced the precision of our morphological evaluations and influenced the observed correlation strengths. Additionally, the assumption of an elliptical tendon shape may not accurately represent tendons with irregular morphology, particularly following rupture. Finally, the imaging technique used prevented accurate length measurements of the lateral gastrocnemius muscle head, potentially limiting the comprehensiveness of our muscle assessments.

## CONCLUSIONS

Our study indicates that tendon elongation alone does not account for low‐functioning outcomes, as elongation was present in both high‐ and low‐functional patients. These findings suggest that additional factors should be considered when aiming to improve muscle performance after rupture. In our cohort, muscle deficits appeared more closely related to soleus muscle morphology and patient age, although these associations should be interpreted with caution. This indicates that soleus‐focused interventions, with tailored pace and load progression, could be beneficial for older patients.

## AUTHOR CONTRIBUTIONS

*Conceptualization and execution*: Pernilla Eliasson and Andreas Meunier. *Imaging analysis*: Pawel Szaro and Katarzyna Bokwa‐Dabrowska. *Interpretation of the results*: Pernilla Eliasson, Katarina Nilsson Helander, Pawel Szaro, and Andreas Meunier. *Writing—original draft preparation*: Pernilla Eliasson. *Funding acquisition*: Pernilla Eliasson. All authors provided critical feedback and helped shape the research, analysis and manuscript.

## CONFLICT OF INTEREST STATEMENT

The authors declare no conflicts of interest.

## ETHICS STATEMENT

Swedish Ethical Review Authority (Dnr: 2019‐05547, with an amendment 2024‐04118‐02. All participants received oral and written information about the study and provided written informed consent.

## Supporting information

Table S1. Functional tests on the uninjured and injured side and LSI in the high‐ and low‐functioning groups.
Table S2. Agreement in measurements between raters.

## Data Availability

The data that support the findings of this study are available on request from the corresponding author. The data are not publicly available due to privacy or ethical restrictions.
