# Supplementary material for: Tendon elongation in the free tendon is evident in patients with and without persistent muscle weakness following an Achilles tendon rupture
Source: Knee Surg Sports Traumatol Arthrosc. 2026 May 20;34(7):2623–38. doi: 10.1002/ksa.70445 (PMC13327510; doi:10.1002/ksa.70445)
Supplement: Supplementary file 1 — Table S1. Functional tests on the uninjured and injured side and LSI in the high‐ and low‐functioning groups. Table S2. Agreement in measurements between raters. [file KSA-34-2623-s001.docx]

**Supplemental table 1. Functional tests on the uninjured and injured side and LSI in the high- and low-functioning groups.**

|  | High-functioning group | | | Low-functioning group | | | High vs low |
| --- | --- | --- | --- | --- | --- | --- | --- |
|  | Uninjured | Injured | LSI % | Uninjured | Injured | LSI % | LSI: p-value |
| ROM, cm | 51.4±4.68 | 53.4±4.47 | 104±9.09 | 54.6±5.50 | 53.7±3.73 | 98.9±7.32 | 0.097 |
| Jump test, cm | 138±21.2 | 136±22.0 | 98.6±7.09 | 115±24.0* | 107±25.5* | 92.5±8.39 | 0.058 |
| Number heel-rises | 27.3±8.20 | 26.4±8.35 | 96.9±8.41 | 29.7±15.3 | 6.00±5.77* | 20.7±14.6 | <0.001 |
| Mean height, cm | 10.1±1.30 | 8.71±1.43 | 86.6±8.04 | 8.66±1.52* | 4.83±2.76* | 54.8±31.3 | <0.001 |
| Max height, cm | 12.1±1.37 | 10.6±1.47 | 88.4±9.60 | 10.1±1.92 | 5.66±2.84* | 56.4±25.6 | <0.001 |
| Total height, cm | 276±98.2 | 229±84.6 | 84.1±13.6 | 254±139 | 38.7±42.1* | 15.0±11.4 | <0.001 |
| Work, J | 2130±891 | 1760±751 | 84.6±13.8 | 2050±1050 | 314±330* | 14.9±11.4 | <0.001 |

*LSI: limb symmetry index (injured side/uninjured side x 100 and expressed as percentage). * Indicates a significant difference between the High- and low-functioning group on the equivalent side*. *p-values derives from Students t-test between the groups.*

**Supplemental table 2. Agreement in measurements between raters.**

| Variable | ICC | 95% CI Lower | 95% CI Upper | p-value |
| --- | --- | --- | --- | --- |
| AP diameter of the Achilles tendon | 0.918 | 0.894 | 0.999 | <0.001 |
| ML diameter of the Achilles tendon | 0.884 | 0.652 | 0.966 | 0.004 |
| Free tendon length | 0.864 | 0.817 | 0.985 | <0.001 |
| Total tendon length | 0.886 | 0.868 | 0.994 | <0.001 |
| Soleus muscle length | 0.895 | 0.889 | 0.998 | <0.001 |
| Soleus muscle AP diameter | 0.883 | 0.823 | 0.991 | <0.001 |
| Soleus muscle ML diameter | 0.875 | 0.841 | 0.989 | <0.001 |

*ICC: intraclass correlation coefficients, CI: Confidence interval,* *AP: anterior-posterior, and ML: mediolateral*
